# High‐frequency oscillatory ventilation versus conventional ventilation: hemodynamic effects on lung and heart

**DOI:** 10.1002/phy2.259

**Published:** 2014-03-26

**Authors:** Andrea Smailys, Jamie R. Mitchell, Christopher J. Doig, John V. Tyberg, Israel Belenkie

**Affiliations:** 1 Department of Cardiac Sciences, University of Calgary, Calgary, Alberta, Canada; 2 Department of Physiology & Pharmacology, University of Calgary, Calgary, Alberta, Canada; 3 The Libin Cardiovascular Institute of Alberta, University of Calgary, Calgary, Alberta, Canada; 4 Faculty of Medicine & Dentistry, Department of Physiology, University of Alberta, Edmonton, Alberta, Canada; 5 Department of Critical Care Medicine, University of Calgary, Calgary, Alberta, Canada; 6 Department of Medicine, University of Calgary, Calgary, Alberta, Canada

**Keywords:** Pulmonary vascular conductance, LV preload, performance

## Abstract

High‐frequency oscillatory ventilation (HFOV) may improve gas exchange in patients who are inadequately ventilated by conventional mechanical ventilation (CV); however, the hemodynamic consequences of switching to HFOV remain unclear. We compared the effects of CV and HFOV on pulmonary vascular conductance and left ventricular (LV) preload and performance at different airway and filling pressures. In anesthetized dogs, we measured LV dimensions, aortic and pulmonary artery (PA) flow, and mean airway (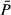
_AW_) and pericardial pressures. Catheter‐tip pressure manometers measured aortic, LV, left atrial, and PA pressures. The pericardium and chest were closed. At LV end‐diastolic pressure (*P*_LVED_) = 5 mmHg and 12 mmHg, PEEP was varied (6 cm H_2_O, 12 cm H_2_O, and 18 cm H_2_O) during CV. Then, at airway pressures equal to those during CV, HFOV was applied at 4 Hz, 10 Hz, and 15 Hz. Increased 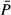
_AW_ decreased pulmonary vascular conductance. As cardiac output increased, conductance increased. At *P*_LVED _= 12 mmHg, conductance was greatest during HFOV at 4 Hz. LV preload (i.e., *A*_LV_, our index of end‐diastolic volume) was similar during HFOV and CV for all conditions. At *P*_LVED _= 12 mmHg, SW_LV_ was similar during CV and HFOV, but, at *P*_LVED _= 5 mmHg and 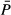
_AW_ 10 cm H_2_O, SW_LV_ was lower during HFOV than CV. Compared to pulmonary vascular conductance at higher frequencies, at *P*_LVED _= 12 mmHg, conductance was greater at HFOV of 4 Hz. Effects of CV and HFOV on LV preload and performance were similar except for decreased SW_LV_ at *P*_LVED _= 5 mmHg. These observations suggest the need for further studies to assess their potential clinical relevance.

## Abstract (precis)

We found that conventional and high‐frequency oscillatory ventilation (HFOV) have similar effects on LV preload and performance except for worse LV performance at a low filling pressure. We also found that pulmonary vascular conductance was greater during high‐frequency oscillatory at 4 Hz at LV end‐diastolic pressure of 12 mmHg compared to at higher frequencies.

## Introduction

Conventional mechanical ventilation (CV) may be insufficient to maintain adequate gas exchange in seriously ill patients and may also cause or aggravate lung injury. High‐frequency oscillatory ventilation (HFOV) has been used to improve gas exchange in patients who cannot be adequately ventilated by conventional means, while putatively limiting lung injury (Heuer et al. 2012; Ip and Mehta 2012; Ferguson et al. 2013). Theoretically, HFOV achieves the goals of protective ventilation by optimizing alveolar recruitment through sustained high mean airway pressures, while avoiding lung trauma associated with the swings in pressure associated with conventional methods (Fort et al. 1997; Mehta et al. 2001, 2004; Derdak et al. 2002). Recent clinical publications suggest that relatively early switching to HFOV may result in no outcome benefit (Young et al. 2013) or might cause harm (Ferguson et al. 2013) so that indications for the use of HFOV will require further refinement.

Although HFOV is used frequently in intensive care units, there are few systematic studies of its hemodynamic effects. The results of studies using different experimental models have not been consistent so it remains still unclear as to what, if any, hemodynamic consequences should be anticipated when switching from CV to HFOV (Fort et al. 1997; Andersen et al. 2002; Derdak et al. 2002; David et al. 2004; Mehta et al. 2004; Nakagawa et al. 2007; Heuer et al. 2012).

A better understanding of the hemodynamic effects of HFOV would facilitate clinical decision making. We therefore performed a canine study at different airway and filling pressures to compare the effects of CV and HFOV. We focused on pulmonary vascular conductance (i.e., the amount of flow that the lungs will accept per unit driving (blood) pressure) and left ventricular (LV) preload and systolic performance.

## Methods

The experimental protocol was approved by the University of Calgary animal care committee, whose criteria are consistent with those of the American Physiological Society.

### Animal preparation

Ten mongrel dogs of both sexes, weighing 15–23 kg (mean 20.3 kg), were anesthetized initially with 25 mg/kg thiopental sodium i.v. (Abbott Laboratories, Montreal, PQ) and a 5 mg/mL i.v. bolus of midazolam (Sandoz, Boucherville, PQ) and were maintained with fentanyl citrate (0.04 mg/mL i.v., initially, followed by an infusion of 4 mg/h, prepared locally), which was adjusted as necessary to ensure deep sedation without spontaneous respiratory effort. The animals were intubated with a cuffed endotracheal tube (9 mm diameter) and ventilated initially with a constant‐volume respirator (model 607, Harvard Apparatus, Mills, MA) with a 1:1 mixture of O_2_ and nitrous oxide. Tidal volume and respiratory rate were initially set at 17 mL/kg and 18 breaths/min, respectively (in accordance with recommended ventilation parameters for large animals (Pascoe 1986)), and were adjusted to maintain physiological blood gas tensions and pH.

### Instrumentation

A median sternotomy was performed with the animals in the supine position. For instrumentation, the heart was delivered from the pericardium via a base‐to‐apex incision. Sonomicrometry crystals (Sonometrics Corp., London, ON) were implanted in the LV endocardium and midwall of the septum to measure minor‐axis septum‐to‐LV free wall (*D*_SLVFW_) and LV anterioposterior (*D*_LVAP_) dimensions (Mitchell et al. 2005a,b). Ultrasonic flow probes (Transonic Systems, Ithaca, NY) were placed on the ascending aorta and main pulmonary artery. Airway pressure (*P*_AW_) was measured at a port on the adapter attached to the proximal end of the endotracheal tube with an air‐filled tube (2 mm diameter) connected to a pressure transducer (model, P23 ID; Statham Gould, Oxnard, CA). A single‐lead electrocardiogram (ECG) was recorded. A flat, fluid‐filled balloon transducer (Smiseth et al. 1986), connected to a pressure transducer (model P23 ID; Statham Gould, Oxnard, CA), was loosely attached to the lateral surface of the LV to measure pericardial pressure (*P*_PERI_).

Catheter‐tip pressure manometers with fluid‐filled reference lumens (7‐F, Millar Instruments Inc., Houston, TX) were used to measure aortic pressure (*P*_Ao_) (inserted through the right femoral artery) and LV pressure (*P*_LV_) (inserted through a carotid artery). Catheter‐tip pressure manometers (3.5‐F, model SPR‐524, Millar Instruments Inc., Houston, TX) were used to measure left atrial (*P*_LA_) (inserted through the left atrial appendage), and pulmonary artery (*P*_PA_) (inserted retrograde through a distal pulmonary artery branch) pressures. An intravenous line was placed in the left external jugular vein for volume loading (Pentaspan™ [10% pentastarch in 0.9% sodium chloride], Bristol‐Myers‐Squibb, St. Laurent, PQ). A left femoral arterial line was used to obtain samples for blood gas analysis (Nova Biomedical, Critical Care Express). The right atrium was paced slightly faster than the intrinsic rate (Grass S88 Stimulator, Grass Instruments, Quincy, MA) to maintain a constant heart rate and to be able to compare hemodynamics without confounding differences in heart rate. Body temperature was measured with a rectal or vaginal thermometer. After instrumentation, the heart was returned to the pericardium, which was closed with single interrupted sutures to approximate normal constraint (Scott‐Douglas et al. 1991). The chest was closed under suction (~ 5 mmHg). An alternative ventilator (Servo, Siemens‐Elema 900C) that enables precise application of PEEP was then connected; 100% O_2_ was delivered throughout the experimental protocol.

### Experimental protocol

After stabilization at a LV end‐diastolic pressure (*P*_LVED_) of 5 mmHg, positive end‐expiratory pressures (PEEP) of 6 cm H_2_O, 12 cm H_2_O, and 18 cm H_2_O were applied in random order during CV and the mean airway pressure (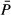
_AW_) at each level of PEEP was noted. All interventions were initiated only after return to baseline with stable hemodynamics for 3–5 min. HFOV (Sensormedics 3100B, Viasys Healthcare, Burlington, ON) was then begun with frequencies of 4 Hz, 10 Hz, and 15 Hz applied to cover a wide, relevant range in randomized order. During ventilation at each frequency, each of the three levels of 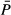
_AW_ noted during CV was applied in randomized order. Once acceptable blood gases were achieved (by adjusting the tidal‐volume amplitude), data were recorded for 1 min. The protocol was then repeated at a *P*_LVED_ of 12 mmHg (Pentaspan™ was infused until the desired *P*_LVED_ was achieved). At the end of the experiment, the animals were sacrificed by an intravenous bolus KCl injection and the positions of the crystals were verified.

### Data analysis

The conditioned signals were amplified (Sonometrics Corp. Acquisition System, London, ON), passed through a low‐pass filter (100 Hz), and digitized at 200 Hz. The digitized data were analyzed using software developed in our laboratory (CV Works, Advanced Measurements Inc., Calgary, AB). Five CV cycles (or the equivalent amount of time during HFOV), during which the cardiac rhythm was regular, were selected for analysis. Using a low‐pass filter, *P*_PERI_ was variably filtered to achieve a smooth trace.

### Calculations

Transmural LV end‐diastolic pressure (*P*_LVEDtm_) = *P*_LVED_ − *P*_PERI_

Cardiac output = SV × heart rate

Pulmonary vascular conductance (i.e., the inverse of pulmonary vascular resistance) = (CO/[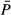
_PA_ − 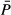
_LA_])

LV stroke work (SW_LV_) = LV stroke volume (SV_LV_) × (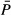
_LV_ (systolic) − 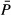
_LA_)

Where, 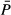
_LV_ (systolic) = *P*_Ao_ (diastolic) + 2⁄3 [*P*_Ao_ (systolic) − *P*_Ao_ (diastolic)]

LV area (*A*_LV_) = *D*_SLVFW _× *D*_LVAP_

*A*_LV_ was used as an index of LV end‐diastolic volume, that is, LV preload (Suga and Sagawa 1974; Appleyard and Glantz 1990). To account for different ventricular dimensions and outputs among animals, selected measurements were normalized with the values at a *P*_LVED_ of 5 mmHg and PEEP of 6 cm H_2_O set as 100%.

### Statistical analysis

The Student's unpaired *t*‐test was used to test for significant changes between CV and HFOV, for a given set of conditions. A *P* value <0.05 was considered statistically significant. Linear regression analysis was used to find the line of best fit for CV and HFOV at each filling pressure. The slopes and Y‐intercepts of these lines were compared using multivariate regression analysis. Using individual values from each condition in each experiment, 95% confidence intervals of the regression line were calculated.

## Results

All data are shown as mean values (± SE's). At a *P*_LVED_ of 5 mmHg, three of the 10 dogs did not tolerate a PEEP of 12 cm H_2_O and five dogs did not tolerate a PEEP of 18 cm H_2_O; they became hemodynamically unstable (systolic *P*_Ao _< 50 mmHg). Mean tidal volume was 18.1 mL/kg (range 16–24 mL/kg) and mean respiratory rate was 17.7 breaths/min (range 17–18 breaths/min). Five of the 10 animals died before the completion of the protocol; thus, at the highest filling pressure, *n* = 5.

Table 1 lists mean airway pressures and the hemodynamic responses during CV (PEEP 6 cm H_2_O) and HFOV (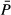
_AW_ ~ 10 cm H_2_O) at *P*_LVED_ 5 mmHg and 12 mmHg. LV preload (*A*_LV_) was affected similarly during both methods of ventilation. However, at a *P*_LVED_ of 5 mmHg, LV performance (SV_LV_ and SW_LV_) was substantially lower during HFOV compared to during CV. At a *P*_LVED_ of 12 mmHg, the difference in LV performance was not statistically significant.

**Table 1.** Baseline hemodynamics at low airway pressure (PEEP 6 cm H_2_O) and both LV filling pressures during CV and HFOV.

*P*_LVED_ 5 mmHg	CV	4 Hz	10 Hz	15 Hz
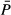 _AW_ (cm H_2_O)	9.7 ± 0.3 (10)	9.1 ± 0.4 (9)	9.0 ± 0.4(10)	^*^8.7 ± 0.3 (10)
HR (beats/min)	98 ± 4.2 (10)	107 ± 3.5 (8)	106 ± 3.1(9)	107 ± 3.1 (9)
*A*_LV_ (mm^2^)	1200 ± 130 (10)	1133 ± 139 (8)	1167 ± 127 (9)	1193 ± 129 (9)
*P*_LVEDtm_ (mmHg)	1.4 ± 0.6 (5)	0.9 ± 0.4 (6)	1.1 ± 0.4 (6)	1.7 ± 0.4 (6)
*P*_LVED_ (mmHg)	6.7 ± 0.5 (8)	6.1 ± 0.6 (8)	6.5 ± 0.6 (9)	6.8 ± 0.7 (9)
mP_LV_(sys) (mmHg)	89 ± 4.4 (8)	87 ± 4.5(8)	81 ± 4.5 (9)	82 ± 3.7 (9)
*P*_Ao_ (ps) (mmHg)	98 ± 4.5 (9)	96 ± 5.0 (8)	91 ± 5.0 (9)	92 ± 4.2 (9)
SV_LV_ (mL)	16.8 ± 2.1(10)	11.9 ± 1.1(9)	^*^12.0 ± 0.9 (10)	12.5 ± 1.0 (10)
SW_LV_ (mL)	1568 ± 170 (8)	^*^1015 ± 114 (8)	^*^945 ± 84 (9)	^*^974 ± 83 (9)
CO (L/min)	1.6 ± 0.2 (10)	1.3 ± 0.1 (9)	1.3 ± 0.1 (10)	1.3 ± 0.1 (10)
*P*_LVED_ 12 mmHg	CV	4 Hz	10 Hz	15 Hz
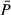 _AW_ (cm H_2_O)	9.4 ± 0.2 (6)	8.4 ± 0.7 (5)	9.0 ± 0.6 (6)	8.4 ± 0.5 (6)
HR (beats/min)	108 ± 6.8 (5)	103 ± 8.5 (4)	104 ± 9.3 (6)	107 ± 11.2(5)
A_LV_ (mm^2^)	1314 ± 220 (5)	1147 ± 166 (4)	1286 ± 177 (6)	1311 ± 209 (5)
*P*_LVEDtm_ (mmHg)	3.1 ± 1.5 (4)	3.8 ± 0.7 (4)	4.0 ± 1.3 (4)	4.0 ± 0.9 (4)
*P*_LVED_ (mmHg)	13.9 ± 0.9 (5)	11.6 ± 0.7 (4)	14.1 ± 1.1 (5)	13.0 ± 0.6 (5)
m*P*_LV_(sys) (mmHg)	93 ± 3.4 (5)	89 ± 3.1 (4)	85 ± 3.1 (5)	^*^90 ± 1.5 (5)
*P*_Ao_ (ps) (mmHg)	103 ± 4.0 (5)	99 ± 3.9 (4)	96 ± 3.5 (5)	95 ± 2.3 (5)
SV_LV_ (mL)	17.2 ± 3.0 (6)	18.2 ± 3.2 (5)	17.1 ± 2.8 (6)	17.5 ± 3.0 (6)
SW_LV_ (mL)	1518 ± 273 (5)	1583 ± 226 (*n* = 4)	1367 ± 198 (*n* = 5)	1371 ± 202 (5)
CO (L/min)	1.8 ± 0.2 (6)	1.8 ± 0.3 (*n* = 5)	1.7 ± 0.2 (*n* = 6)	1.8 ± 0.2 (6)

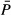
_AW_, mean airway pressure; HR, heart rate; A_LV,_ LV area; *P*_LVEDtm,_ transmural LV end‐diastolic pressure; *P*_LVED_, LV end‐diastolic pressure; LV(sys), mean LV systolic pressure; *P*_Ao_(ps), aortic systolic pressure; SV_LV_, LV stroke volume; SW_LV_, LV stroke work; CO, cardiac output.

*P < .05: *n* is shown in brackets.

**Figure 1.**
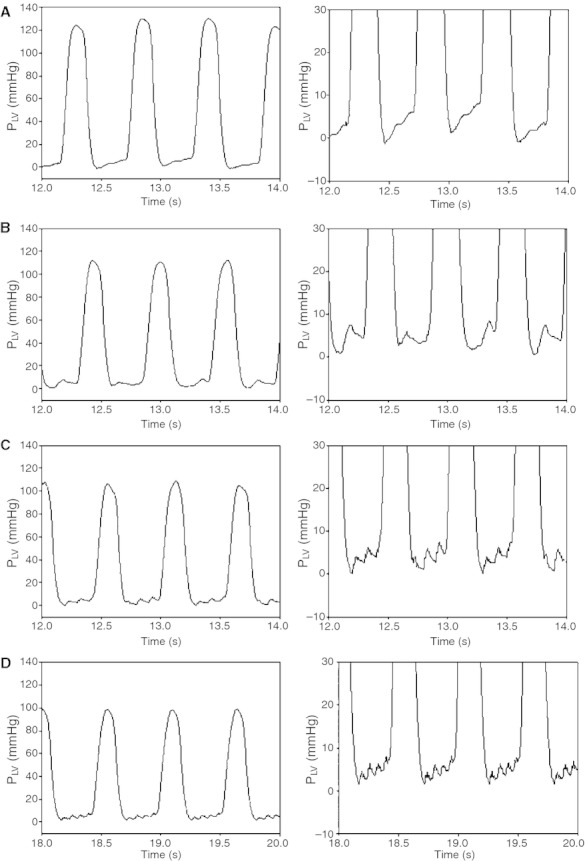
Representative examples of LV pressure traces (mmHg) at *P*_LVED_ 5 mmHg and PEEP 6 cm H_2_O during CV and HFOV (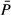
_AW_ ~ 10 cm H_2_O). Panel A is an example of LV pressure during CV. Panels B, C, and D are examples of LV pressure during HFOV 4 Hz, 10 Hz, and 15 Hz, respectively. The vertical scales on the left range are −10 mmHg to 140 mmHg and those on the right are from −10 mmHg to 30 mmHg.

Figure 1 shows typical LV pressure traces during CV and HFOV at a *P*_LVED_ of 5 mmHg and 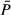
_AW_ ~ 10 cm H_2_O. The LV diastolic pressure trace during CV is characteristic of normal physiological changes during diastole. During HFOV, note the fluctuations in LV diastolic pressure due to the oscillations in airway pressure. No fluctuations are apparent during LV systolic pressure.

### Pulmonary vascular conductance

**Figure 2.**
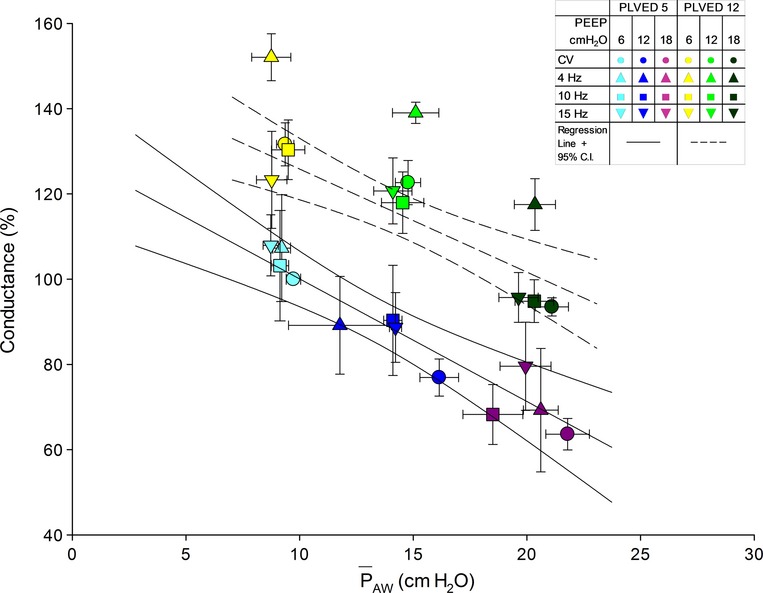
Relations between normalized pulmonary vascular conductance and 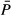
_AW_ during CV and HFOV. The solid lines illustrate the best‐fit linear regression and 95% confidence intervals for all the data at *P*_LVED_ 5 mmHg. The dashed lines indicate the best‐fit linear regression and 95% confidence intervals for all the data at *P*_LVED_ 12 mmHg, except for HFOV at 4 Hz.

The relations between pulmonary vascular conductance and 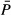
_AW_ during CV and HFOV at *P*_LVED_ of 5 mmHg and 12 mmHg are illustrated in Figure 2. At both filling pressures, as 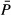
_AW_ increased, conductance substantially decreased. At a *P*_LVED_ of 5 mmHg, conductance decreased by ~40% when 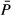
_AW_ was increased from ~10 cm H_2_O to ~20 cm H_2_O. This decrease in conductance would seem to be a “cost” of increasing 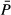
_AW_, regardless of the mode of ventilation. As CO increased (here, due to an increase in *P*_LVED_), conductance also increased (note that all the yellow and green symbols lie above their respective blue and purple symbols). This suggests that the lung adapts to increases in CO, increasing conductance by “recruitment” of pulmonary vasculature. When *P*_LVED_ was increased from 5 mmHg to 12 mmHg, note that conductance increased by ~50% at 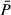
_AW_ ~15 cm H_2_O. It is potentially important that at a *P*_LVED_ of 12 mmHg, HFOV at a frequency of 4 Hz seems to be particularly advantageous. At 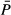
_AW_ ~15 cm H_2_O, conductance was ~15% higher at HFOV 4 Hz than at the higher frequencies; this apparent 4‐Hz “advantage” was not observed at *P*_LVED_ 5 mmHg.

**Figure 3.**
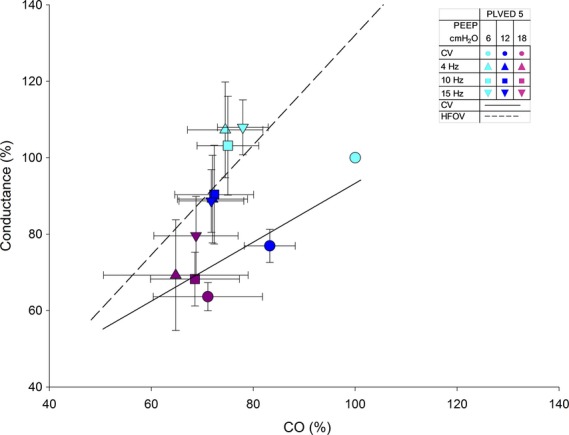
Relations between normalized pulmonary vascular conductance and normalized CO for *P*_LVED_ 5 mmHg. The solid line is the best‐fit linear regression of the CV data and the dashed line, of the HFOV data. The slopes of the HFOV and CV data are statistically different (*P *<**0.001).

Figure 3 shows how HFOV appears to augment pulmonary vascular conductance at a *P*_LVED_ of 5 mmHg. The HFOV data lie to the left of the CV data such that a normalized conductance of 100% is achieved at a CO of ~70% with HFOV, rather than a CO of 100% with CV. As indicated in Figure 2, conductance generally increases as CO increases, as the lung accommodates by recruitment. However, note the apparent advantage of HFOV where, as CO increases, the lung can accept increased CO as compared to CV. There was no such difference between CV and HFOV at a *P*_LVED_ of 12 mmHg.

### Cardiac function during CV and HFOV

**Figure 4.**
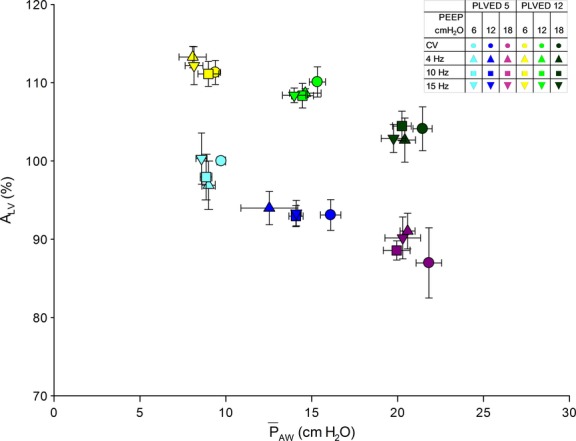
Relations between normalized LV preload (A_LV_) and 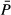
_AW_ during CV and HFOV. At each airway and filling pressure, A_LV_ was similar during both modes of ventilation. Volume loading increased A_LV_ at a given 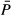
_AW_, and increasing 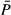
_AW_ decreased A_LV_ at both filling pressures.

**Figure 5.**
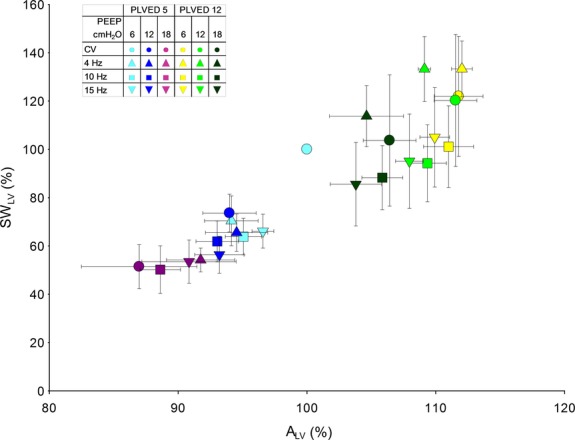
Relations between normalized SW_LV_ and normalized A_LV_ during CV and HFOV. SW_LV_ was closely related to A_LV_, as predicted by the Frank–Starling mechanism.

The effects of manipulating 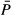
_AW_ and *P*_LVED_ on cardiac performance are shown in Figures 4 and 5. Figure 4 shows how increasing 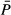
_AW_ reduced LV preload (i.e., *A*_LV_) and how volume loading increased preload. LV preload at each value of 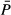
_AW_ and *P*_LVED_ was similar during both modes of ventilation. Figure 5 shows the dependence of SW_LV_ on *A*_LV_. As expected, SW_LV_ increased as *A*_LV_ increased.

**Figure 6.**
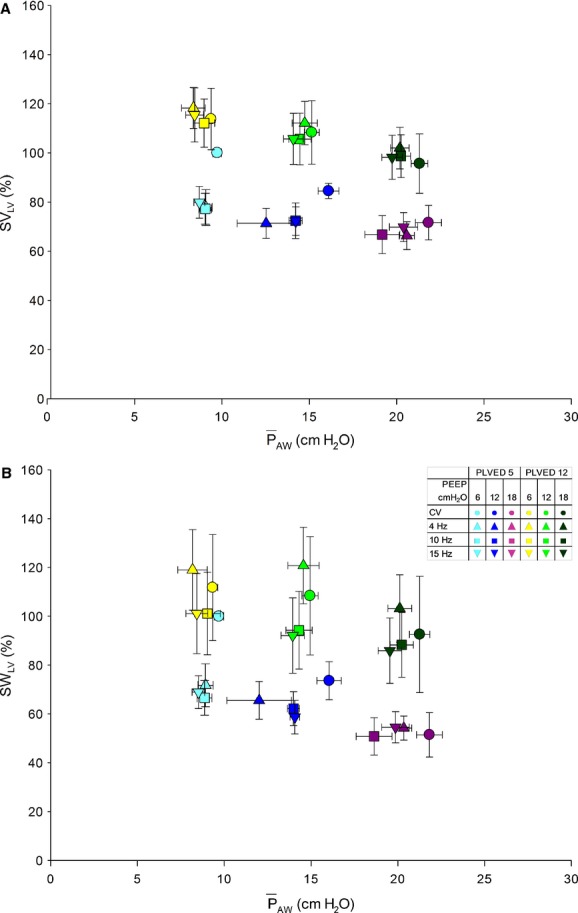
Relations between 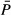
_AW_ and normalized SV_LV_ (A) and normalized SW_LV_ (B) during CV and HFOV. At *P*_LVED_ 12 mmHg, SV_LV_ and SW_LV_ were statistically similar during CV and HFOV for a given 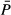
_AW_. At *P*_LVED_ 5 mmHg and 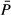
_AW_ ~10 cm H_2_O, SV_LV_ and SW_LV_ were significantly lower during HFOV (all frequencies) than during CV (*P* < 0.001, *P* = 0.001, respectively).

The relations between 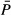
_AW_ and LV performance [normalized SV_LV_ (A) and normalized SW_LV_ (B)] during CV and HFOV are illustrated in Figures 6A and B, respectively. SV_LV_ and SW_LV_ were greater at the higher filling pressure and decreased at higher airway pressures. At a *P*_LVED_ of 12 mmHg and at each 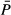
_AW_, SV_LV_ and SW_LV_ were statistically similar during CV and HFOV. However, at the lower filling pressure (*P*_LVED _= 5 mmHg) and 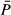
_AW_ ~10 cm H_2_O, SV_LV_ and SW_LV_ were significantly lower during HFOV (all frequencies) than during CV (*P *<**0.001, *P *=**0.001, respectively). The differences were not significant at higher airway pressures.

**Figure 7.**
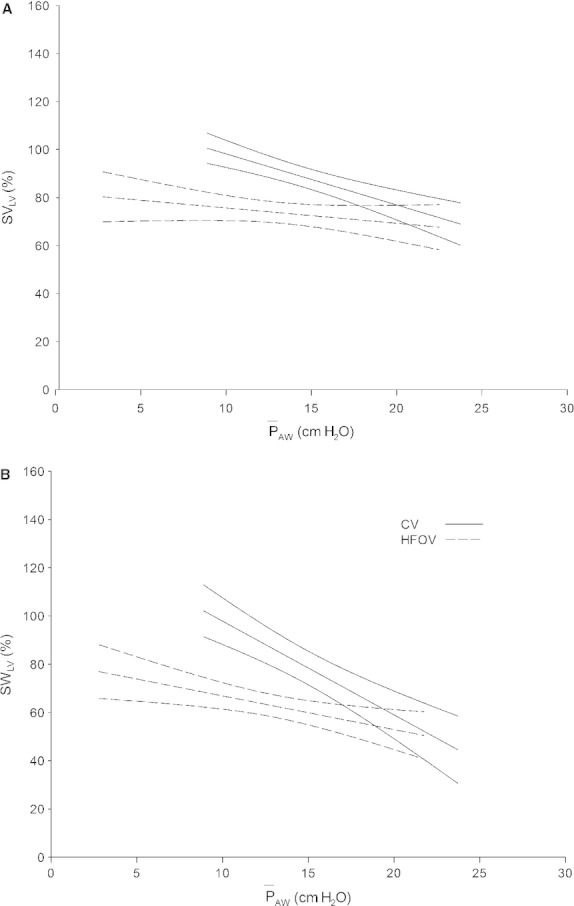
Regression relations of normalized SV_LV_ (A) and normalized SW_LV_ (B) with 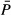
_AW_ measured only at *P*_LVED_ 5 mmHg during CV and HFOV. In both of these relations, it can be seen that at the lower filling pressure, the regression lines for HFOV have a significantly different slope than for CV (both *P* < 0.01) and a significantly different Y‐intercept (*P* < 0.01 and *P* < 0.001, respectively).

The regression relationships that relate LV performance (normalized SV_LV_ and normalized SW_LV_) to 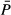
_AW_ during CV and HFOV are shown in Figures 7A and B, respectively. Note that these regressions are only for data obtained at a *P*_LVED_ of 5 mmHg. In both Figures 7A and B, the regression lines for HFOV have a significantly different slope than those for CV (*P *<**0.01 for both) and a significantly different Y‐intercept (*P *<**0.01 and *P *<**0.001, respectively), which suggests that LV performance is reduced more during HFOV than during CV at low filling pressures.

**Figure 8.**
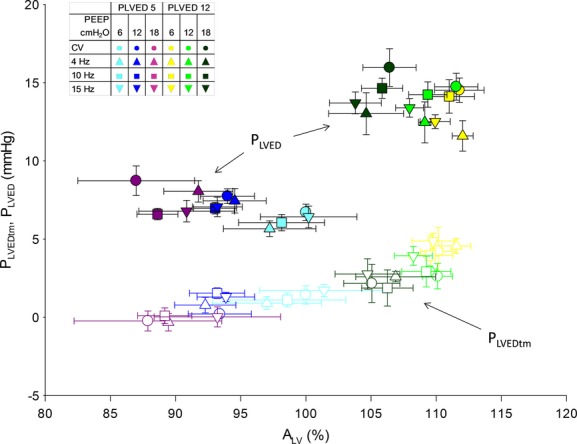
The predictable relations between *P*_LVED_, *P*_LVEDtm_, and A_LV_ over the range of filling pressures and positive end‐expiratory pressures are apparent.

The relations between *P*_LVED_, *P*_LVEDtm_, and *A*_LV_ are shown in Figure 8. Increased PEEP increased LV intracavitary pressure which might suggest increased LV preload. However, after accounting for external constraint, it was shown that preload (*A*_LV_) increased as a quasi‐sigmoidal function of *P*_LVEDtm_. The scatter in that relationship was minimal and the sigmoidal shape was expected, the LV being a structure with an unstressed volume that becomes stiffer as volume increases.

Table 2 lists normalized RV stroke work (SW_RV_) and SW_LV_ during CV (all PEEP levels) and HFOV (all frequencies and matched airway pressures) (100% set at prevolume load, CV PEEP 6 cm H_2_O). At *P*_LVED_ = 5 mmHg, both SW_RV_ and SW_LV_ decreased during CV with increasing airway pressure; however, during HFOV, while SW_LV_ decreased, SW_RV_ generally increased with increased 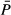
_AW_. This reciprocal relationship may explain the apparent differences in LV performance during the two modes of ventilation – see Instrumentation. At the higher filling pressure, both SW_RV_ and SW_LV_ decreased with increasing airway pressure during both CV and HFOV (except at 10 Hz, 20 cm H_2_O 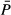
_AW_).

**Table 2.** Normalized RV and LV stroke work

	*P*_LVED_ 5 mmHg		*P*_LVED_ 12 mmHg	
	SW_RV_	SW_LV_	SW_RV_	SW_LV_
PEEP 6 cm H_2_O	100 ± 0 (7)	100 ± 0 (8)	74 ± 14.3 (4)	112 ± 21.8 (5)
PEEP 12 cm H_2_O	83 ± 9.1 (5)	74 ± 8.6 (6)	72 ± 17.2 (4)	108 ± 24.3 (5)
PEEP 18 cm H_2_O	65 ± 4.8 (4)	51 ± 9.1 (5)	70 ± 17.2 (4)	93 ± 23.8 (5)
4 Hz; 10 cm H_2_O	62 ± 7.2 (7)	72 ± 8.8 (7)	85 ± 16.6 (3)	119 ± 16.5 (4)
4 Hz; 15 cm H_2_O	62 ± 8.7 (5)	66 ± 7.7 (6)	83 ± 16.4 (3)	121 ± 15.7 (4)
4 Hz; 20 cm H_2_O	72 ± 9.7 (3)	54 ± 4.9 (5)	82 ± 17.1 (3)	103 ± 13.8 (4)
10 Hz; 10 cm H_2_O	67 ± 8.4 (8)	67 ± 7.0 (8)	71 ± 11.9 (4)	101 ± 16.9 (5)
10 Hz; 15 cm H_2_O	68 ± 10 (5)	62 ± 6.9 (6)	71 ± 11.8 (4)	94 ± 15.9 (5)
10 Hz; 20 cm H_2_O	66 ± 14.2 (4)	51 ± 7.6 (5)	75 ± 11.7 (4)	88 ± 13.3 (5)
15 Hz; 10 cm H_2_O	68 ± 7 (8)	69 ± 6.7 (8)	81 ± 9.9 (4)	101 ± 16.5 (5)
15 Hz; 15 cm H_2_O	63 ± 10 (6)	59 ± 6.9 (7)	75 ± 7.8 (4)	92 ± 15.4 (5)
15 Hz; 20 cm H_2_O	70 ± 14 (3)	55 ± 6.4 (4)	75 ± 7.0 (4)	86 ± 13.4 (5)

SW_RV_, RV stroke work; SW_LV_, LV stroke work.

## Discussion

In this study, we made two important observations. First, we observed that pulmonary vascular conductance, the amount of flow that the lungs will accept per unit driving (blood) pressure, varied under the different experimental conditions to a degree that may be clinically important. Second, we found that LV performance was similar during CV and HFOV at similar airway pressures, consistent with the fact that LV preload was not changed by switching to from CV to HFOV.

Since the lung is a very complicated hydraulic‐pneumatic system, ventilation may have profound phase‐specific effects on cardiac and lung function through a number of complex and interactive mechanisms (Morgan et al. 1966). These effects are primarily through changes in lung volume and intrathoracic pressure (Pinsky 1997). Given the very different patterns of changes in airway and intrathoracic pressure with the two modes of ventilation, it is not surprising that hemodynamic differences may exist between CV and HFOV. The cyclic changes in airway pressure during CV have considerably different effects on changes in LV and RV preload (and therefore performance) (Mitchell et al. 2005a) than those that would be expected during HFOV, which should resemble the effects observed with variations in PEEP, yet lack the cyclic effects associated with CV.

### Effects of mechanical ventilation on pulmonary vascular conductance

A major objective of this study was to determine the effects of HFOV on the pulmonary vasculature of the lung. It can be argued that pulmonary vascular conductance is a better and more appropriate measure of the vascular properties of the lung than the common measure, pulmonary vascular resistance (PVR). Conductance is normalized flow – the rate of blood flow that an organ will accept at a given driving pressure (i.e., conventionally, the difference between mean arterial and venous pressures).

The observed changes in pulmonary vascular conductance are consistent with previously reported work (Mitchell et al. 2005a). Increased airway pressure, presumably through compression of the pulmonary vessels, increased resistance and decreased conductance. Volume loading increased conductance, presumably through recruitment of pulmonary vessels and distension of the already recruited vessels, thereby reducing the effects of external compression due to increased lung volume (Fig. 2) (Mitchell et al. 2005b; West 2006). At the higher filling pressure, it is interesting and perhaps important that HFOV at 4 Hz appears to confer a special advantage in terms of increased pulmonary vascular conductance suggesting frequency‐dependent effects on lung conductance. We cannot explain the increased conductance at 4 Hz. However, the magnitude of the increased conductance may represent a clinically relevant hemodynamic advantage. Additional studies would be required to determine what the optimal range of HFOV frequencies are, particularly in patients with unstable hemodynamics.

Cardiac output and conductance are linearly related (Fig. 3), which indicates that the pulmonary vasculature accepts an increase in flow without a proportional increase in the pressure gradient (Bouwmeester et al. 1985).

High‐frequency oscillatory ventilation had a noticeable advantage on pulmonary vascular conductance at the lower filling pressure (*P*_LVED_ 5 mmHg); as cardiac output increased, the lung was able to accept increased output (Fig. 3)**.** Interestingly, there was no such effect at the higher filling pressure, *P*_LVED_ 12 mmHg. This suggests that most or all pulmonary vessels were already recruited at the higher filling pressure.

The “chicken versus egg” debate between conductance and cardiac output still remains. Is decreased conductance the primary response to increased airway pressure (due to compression of the pulmonary vessels) and cardiac output decreases as a result? Or, is decreased cardiac output the primary response to increased airway pressure (due to external cardiac constraint) and conductance decreases as a result? Our study does not resolve this issue but demonstrates that regardless of the mechanism, the linear relationship differs between the two modes of ventilation at the lower filling pressure (Fig. 3).

### Effects of mechanical ventilation on LV preload

The other major objective of this study was to determine if switching from conventional to high‐frequency oscillatory ventilation at similar airway pressures would have similar effects on LV preload. Increased intrathoracic pressure may limit diastolic filling by several mechanisms. These include increased external constraint to the heart (Haynes et al. 1980; Marini et al. 1981; Cassidy and Ramanathan 1984; Kingma et al. 1987), redistribution of blood from the thorax to the periphery, and direct ventricular interaction (Cassidy and Mitchell 1981; Cassidy and Ramanathan 1984; Gibbons Kroeker CA 2003; Mitchell et al. 2005a,b). Importantly, our data suggest that the effects of mechanical ventilation on LV preload (i.e., end‐diastolic *A*_LV_) are closely related to mean airway and filling pressures and not to the mode of ventilation (Fig. 4). Thus, during both CV and HFOV, LV preload decreased similarly with increased airway pressure, and increased with volume loading, which also limited the decrease in LV preload associated with increased airway pressure. As one might predict in these acute studies, LV performance (SV_LV_ and SW_LV_) was similar and closely related to LV preload during both modes of ventilation, except, as discussed below, at the lower filling pressure (Haynes et al. 1980; Marini et al. 1981; Linderer et al. 1983; Belenkie et al. 1989).

### Effects of mechanical ventilation on LV performance

The relationship between LV preload (defined as LV end‐diastolic area or transmural pressure) and systolic performance (LV stroke work) has been remarkably predictive during acute experiments in which the hemodynamic effects of mechanical ventilation have been studied (Mitchell et al. 2005a, 2011). Although this was generally true in this study as well, LV performance appeared to be adversely affected during HFOV compared to CV at low LV filling pressure (*P*_LVED_ 5 mmHg) and PEEP 6 cm H_2_O (Fig. 6), while these differences were not apparent with increased filling and airway pressures. We recognize that clinically, there are strong arguments to avoid low filling pressures in ventilated patients. Nevertheless, it remains interesting that there is a difference in LV performance between the two modes of ventilation under those conditions. Heuer et al. (2012) similarly found no hemodynamic differences between CV and HFOV with increased airway pressure and demonstrated that, at a LV filling pressure of 12 mmHg, HFOV was associated with less adverse hemodynamic effects.

### Decreased LV performance at low filling pressure

Positive end‐expiratory pressures may increase the RV end‐diastolic volume with both a decreased *D*_LVAP_ and a leftward septal shift (and flattening) through direct ventricular interaction. We and others have shown that this is more likely at low filling pressures (Jardin et al. 1981; Mitchell et al. 2005a,b). This may explain the apparent differences in LV performance between CV and HFOV at the lower filling pressure. Direct ventricular interaction during HFOV should resemble that observed at end‐expiration with PEEP since HFOV delivers a sustained high airway pressure and minimizes swings in airway pressure. There is no apparent difference in LV performance between CV and HFOV at the higher filling pressure when little or no leftward septal shift would be expected (Fig. 5). We speculate that at the low filling pressure, there was septal flattening and less systolic leftward (normal) septal motion and possibly even paradoxic (systolic rightward septal motion). This, in effect, would transfer LV work to the RV. That the decrease in SW_LV_ was generally associated with an increase in SW_RV_ (Table 2) is consistent with the suggested mechanism. Paradoxic septal motion presumably decreased LV performance by decreasing the septal contribution to LV output while increasing the septal contribution to RV performance through transfer of pressure via the septum. This is similar to what occurs with left bundle branch block where LV dysfunction can be improved when paradoxic septal motion is corrected through cardiac resynchronization pacing.

### Clinical significance

Many hemodynamic variables appear to be affected similarly during CV and HFOV, which provide a degree of confidence to the clinician that switching to HFOV might not be associated with a hemodynamic disadvantage. In any mode of ventilation, high airway pressures and low filling pressures are associated with worse outcomes. Our data our in keeping with the well‐known concepts that LV performance may be decreased at lower LV filling pressures, which can be prevented by insuring adequate filling pressures. In addition, our finding of improved pulmonary vascular conductance at the lower frequency during HFOV deserves further study to determine its potential clinical relevance.

### Limitations

A normal, anesthetized animal model was used in this study. Clinically, HFOV is used in patients with abnormal lungs. Therefore, while our data provide insight into the hemodynamic effects of HFOV in healthy animals, our observations cannot be extrapolated to clinical situations without first performing similar work in appropriate models.

Although 10 dogs were studied, five died before completion of the protocol. The cause(s) of death could not be determined, but the long, complicated protocol, which included high levels of PEEP at a low filling pressure, may have had detrimental effects on the animals. Acidosis was observed in several dogs, especially in earlier experiments, when amplitude adjustments were not adequate to maintain normal CO_2_ and pH levels. However, our data do not show a consistent effect of pH (within our observed ranges: 7.10–7.45) on cardiac function (data not shown).

Left ventricular preload is sometimes difficult to assess. Although LV transmural pressure is an appropriate measure to assess LV preload (Marini et al. 1981; Belenkie et al. 2002), there were challenges with its measurement during HFOV. Large high‐frequency oscillations in airway pressure complicated the measurement of low pressures such as pericardial and diastolic intracavitary pressures. LV area was minimally affected by the rapid pressure oscillations and therefore was relied upon as the measure of LV preload in this study (Suga and Sagawa 1974; Appleyard and Glantz 1990).

## Conclusions

Although increased airway pressure decreases pulmonary vascular conductance (i.e., the blood flow the lung will accept for the same pressure difference) regardless of the mode of ventilation, HFOV at a frequency of 4 Hz appears to increase conductance substantially more than at the higher frequencies we studied. Also, HFOV appears to increase conductance relative to conventional ventilation at any level of cardiac output. These observations suggest that there is a need for further study in appropriate models to assess the potential clinical importance of such differences.

We also conclude that switching from CV to HFOV at similar airway pressures appears to have no significant effect on LV preload and that LV performance should also be unaffected except at low filling pressures, which are generally avoided clinically.

## Acknowledgments

The authors appreciate the outstanding technical assistance of Cheryl Meek and the helpful suggestions of Dr. Andrei Harabor.

## Conflict of Interest

The authors declare no conflict of interest.
